# Childhood medulloblastoma in Britain 1971-77: analysis of treatment and survival.

**DOI:** 10.1038/bjc.1983.274

**Published:** 1983-12

**Authors:** C. A. Stiller, E. L. Lennox

## Abstract

In a population-based series of 368 children undergoing surgery for medulloblastoma, 304 (83%) survived to complete a course of radiotherapy. Among those patients who completed radiotherapy, the short-term survival rates were lower for young children (those aged under 5 years) than for older children, but by 6 years the survival rates were very similar (approximately 35%) for children in both age groups. Higher survival rates were obtained in the young children where total macroscopic excision of the tumour was achieved. For older children there was no difference in survival rates between those with total or partial excision, though the survival rate was lower for those whose surgery was limited to biopsy. In young children radiotherapy dose had no effect on survival rates. In older children, survival rates were appreciably higher where doses had been at least 45 Gy to the posterior fossa and 30 Gy to the spinal cord, and there were also fewer spinal cord metastases among those who received a higher spinal cord dose. Ninety (30%) of the 304 children also received chemotherapy as part of their initially planned treatment; a wide variety of protocols was used and no conclusions could be drawn as to the effects on survival rates.


*Br. J. Cancer* (1983), **48**, 835–841

### Childhood medulloblastoma in Britain 1971–77: Analysis of treatment and survival

C.A. Stiller & E.L. Lennox

*Childhood Cancer Research Group, University of Oxford, Department of Paediatrics, Radcliffe Infirmary, Oxford OX2 6HE*

**Summary** In a population-based series of 368 children undergoing surgery for medulloblastoma, 304 (83%) survived to complete a course of radiotherapy. Among those patients who completed radiotherapy, the short-term survival rates were lower for young children (those aged under 5 years) than for older children, but by 6 years the survival rates were very similar (~35%) for children in both age groups. Higher survival rates were obtained in the young children where total macroscopic excision of the tumour was achieved. For older children there was no difference in survival rates between those with total or partial excision, though the survival rate was lower for those whose surgery was limited to biopsy. In young children radiotherapy dose had no effect on survival rates. In older children, survival rates were appreciably higher where doses had been at least 45 Gy to the posterior fossa and 30 Gy to the spinal cord, and there were also fewer spinal cord metastases among those who received a higher spinal cord dose. Ninety (30%) of the 304 children also received chemotherapy as part of their initially planned treatment; a wide variety of protocols was used and no conclusions could be drawn as to the effects on survival rates.

In the past, medulloblastoma has been generally regarded as having a very poor prognosis. The 3-year and 5-year survival rates for children diagnosed in Great Britain during 1962–70 were only 25% and 18% respectively (Draper *et al.*, 1982). Treatment during this period consisted of surgery, with varying degrees of removal of the tumour, followed by a course of radiotherapy; chemotherapy was sometimes used to treat recurrences. From 1970, at an increasing number of centres, children were also given chemotherapy as part of their initial treatment in addition to surgery and radiotherapy (Bloom, 1979; Marsden & Steward, 1976), and clinical trials were set up to study the effect on survival of maintenance chemotherapy (Berry *et al.*, 1981; Gerosa *et al.*, 1981). The purpose of the present analysis is to examine the possible effects on survival of variations in the initial planned treatment of childhood medulloblastoma.

### Patients and methods

During 1971–77 there were 368 children in Great Britain notified through the national cancer registration scheme with a diagnosis of medulloblastoma which was histologically confirmed following surgery. Confirmation of diagnosis and information on treatment and follow-up were obtained from the hospitals at which the children were treated or from their family doctors. All children who were not already known to have died, and who were resident in England and Wales at the time of diagnosis, were "flagged" at the National Health Service Central Register, so that any deaths would automatically be notified to us, and have thus been followed up to the end of 1982. We have received death certificates for children in Scotland dying of cancer up to the end of 1982; for the purpose of the present analysis we have assumed that children in Scotland not notified to us as having died, were still alive on 31 December 1982. In the series under review there were 239 boys and 129 girls. At the time of operation, 40 of the children were aged under 2 years, 88 were aged 2–4, 151 were aged 5–9 and 89 were aged 10–14.

Correspondence: C.A. Stiller

Received 18 July 1983; accepted 26 September 1983.

### Results

### *Treatment*

Of the 368 children who were ascertained, 47 died post-operatively without receiving further treatment. Sixteen further children died before their radiotherapy could be completed, and another was withdrawn from radiotherapy by his parents. The remaining 304 children received a complete course of radiotherapy. Table I shows the total tumour dose of radiotherapy to the posterior fossa for these 304 children. Doses varied from 15.5 to 55 Gy, with a tendency to give higher doses during the more recent years. Although there were children in both

© The Macmillan Press Ltd., 1983

**Table I** Total dose to posterior fossa in completed course of radiotherapy

| | | *Total dose (Gy)* | | | | | |
|---|---|---|---|---|---|---|---|
| | | *<40* | *40–44* | *45–49* | *50+* | *NR* | *Total* |
| Year of diagnosis | 1971–74 | 39 (23%) | 36 (21%) | 42 (24%) | 51 (29%) | 5 (3%) | 173 |
| | 1975–77 | 21 (16%) | 23 (18%) | 42 (32%) | 44 (34%) | 1 (1%) | 131 |
| Age (years) at diagnosis | | | | | | | |
| | 0–4 | 24 (25%) | 20 (21%) | 33 (34%) | 19 (20%) | 1 (1%) | 97 |
| | 5–14 | 36 (17%) | 39 (19%) | 51 (25%) | 76 (37%) | 5 (2%) | 207 |
| Total | | 60 (20%) | 59 (19%) | 84 (28%) | 95 (31%) | 6 (2%) | 304 |

**Table II** Total dose of radiotherapy to spinal cord

| *Age (years) at diagnosis* | *Posterior fossa dose (Gy)* | *Spinal cord dose (Gy)* *<30* | *30–34* | *35+* | *NR* | *Total* |
|---|---|---|---|---|---|---|
| 0–4 | <45 | 21 (48%) | 11 (25%) | 6 (14%) | 6 (14%) | 44 |
| 0–4 | 45+ | 5 (10%) | 32 (62%) | 10 (19%) | 5 (10%) | 52 |
| 5–14 | <45 | 21 (28%) | 24 (32%) | 11 (15%) | 19 (25%) | 75 |
| 5–14 | 45+ | 15 (12%) | 69 (54%) | 29 (23%) | 14 (11%) | 127 |
| Total | | 62 (21%) | 136 (46%) | 56 (19%) | 44 (15%) | 298 |

age groups who received doses in the lowest and highest ranges, there was a tendency for older children to be given a higher dose. Some of the children later underwent further radiotherapy for recurrence of their tumours: this additional treatment has been excluded from Table I. Table II shows the distribution of radiotherapy doses to the spinal cord for children with a known posterior fossa dose. As with the treatment of the posterior fossa, younger children tended to be given a lower dose. A higher spinal cord dose was associated with a higher posterior fossa dose in children of all ages. The radiotherapy dose to the whole brain has not been separately tabulated. Where it was known, this was the same as the spinal cord dose in 74% of cases, and in the range between spinal cord and posterior fossa dose in the remainder.

A summary of the chemotherapy used in the initial planned treatment of the children who completed a course of radiotherapy is given in Table III. A small number of patients were given chemotherapy during the period between diagnosis and the completion of radiotherapy, with no further treatment thereafter. A larger number received chemotherapy for a period of up to 2 years following radiotherapy; many of these children had also received chemotherapy during the period up to

**Table III** Chemotherapy given to children completing radiotherapy

| *Chemotherapy* | *Age (years) at diagnosis* *0–4* | *5–14* | *Total* |
|---|---|---|---|
| None | 65 | 149 | 214 |
| *Before completion of radiotherapy only* | | | |
| VCR alone | 2 | 4 | 6 |
| MTX alone | 0 | 1 | 1 |
| VCR+MTX | 2 | 1 | 3 |
| **Post-radiotherapy* | | | |
| VCR alone | 2 | 4 | 6 |
| VCR+MTX+CCNU | 1 | 5 | 6 |
| VCR+MTX | 5 | 1 | 6 |
| VCR+BCNU | 6 | 7 | 13 |
| VCR+CCNU | 9 | 22 | 31 |
| VCR+CCNU+PCZ | 3 | 6 | 9 |
| Other | 2 | 7 | 9 |
| Total | 97 | 207 | 304 |

VCR = vincristine.
MTX = methotrexate.
PCZ = procarbazine.
*Some children in this group also received chemotherapy before completion of radiotherapy.

the end of radiotherapy. The "other combinations" group of Table III includes various combinations of vincristine, methotrexate, CCNU, procarbazine, VM26 and DTIC. Some children, including some who had not previously received chemotherapy, were given chemotherapy as treatment for recurrence of their tumours. This chemotherapy has been excluded from Table III. Thirteen children whose tumours were diagnosed during 1971–74 were included in a trial of immunotherapy using killed medulloblastoma vaccine. For the purposes of Table III and all other analyses presented here, these children are included in the "no chemotherapy" group. There was no significant difference between the age groups in the proportion of children receiving chemotherapy.

*Survival*

The overall five-year survival rate for the 368 surgically treated children in this series was 32%. The rate was 27% for those diagnosed during 1971–74 and 38% for 1975–77.

Table IV shows the proportions of children who survived to complete their radiotherapy, classified

**Table IV** Proportions of children surviving to complete a course of radiotherapy classified by year of diagnosis, age and extent of surgery

| *Year of diagnosis* | *No radiotherapy given* | *Radiotherapy started but not completed* | *Radiotherapy completed* | *Total* |
|---|---|---|---|---|
| 1971–74 | 29 | 12 | 173 (81%) | 214 |
| 1975–77 | 18 | 5 | 131 (85%) | 154 |
| Age at diagnosis (years) | | | | |
| 0–4 | 22 | 9 | 97 (76%) | 128 |
| 5–14 | 25 | 8 | 207 (86%) | 240 |
| Extent of surgery | | | | |
| biopsy only | 12 | 3 | 43 (74%) | 58 |
| Subtotal excision | 28 | 11 | 177 (82%) | 216 |
| Total excision | 6 | 2 | 81 (91%) | 89 |
| Unknown | 1 | 1 | 3 | 5 |
| Total | 47 | 17 | 304 (83%) | 368 |

by year of diagnosis, age and extent of surgery. The slight increase in more recent years in the proportion of children completing their radiotherapy was not statistically significant. The proportion of children surviving to complete their radiotherapy was significantly higher among those aged 5–14 years than in the younger age group ($\chi^2=5.66$ on 1 df, $P<0.05$). The remaining survival analyses are based on the 304 children who received a complete course of radiotherapy.

There was no difference in survival rates between the sexes. Figure 1 shows the actuarial survival curves for children aged 0–4 and 5–14 years at diagnosis. There was a significant difference in 3-year survival rates between the age groups ($\chi^2=7.76$ on 1 df, $P<0.01$), but the overall difference between the two survival curves was not statistically significant ($\chi^2=2.97$ on 1 df, $P>0.05$) and by 6 years from diagnosis the two age groups had very similar rates.

**Figure 1** Actuarial survival curves for children aged 0–4 years (——; $n=97$ and 5–14 (----; $n=207$) at diagnosis.

Figure 2 shows the survival curves for children aged under 5 years classified according to whether or not total macroscopic excision of their tumour was achieved. Survival rates in this age group were significantly higher for patients whose tumours were totally removed than for those who had residual tumour present after operation ($\chi^2=4.66$ on 1 df, $P<0.05$). There was no difference in survival among children aged 5–14 years between those whose tumours were totally removed and those whose tumours were only partially removed. The survival rate for children in this age group who underwent biopsy alone was significantly lower than for those who had more extensive surgery ($\chi^2=7.18$ on 1 df, $P<0.01$).

Figure 3 shows the relationship between posterior fossa radiotherapy dose and survival among children aged 5–14 years. Survival rates were significantly higher in those who received at least 45 Gy to the posterior fossa ($\chi^2=16.7$ on 1 df,

**Figure 2** Actuarial survival curves for children aged 0–4 years at diagnosis, classified according to extent of surgical excision of tumour.

**Figure 3** Actuarial survival curves for children aged 5–14 years at diagnosis, classified according to posterior fossa radiotherapy dose.

$P<0.0001$). For children in this age group there was also a significant trend towards higher survival rates with increasing dose of radiotherapy to the spinal cord (Figure 4; $\chi^2=5.17$ on 1 df, $P<0.05$).

**Figure 4** Actuarial survival curves for children aged 5–14 years at diagnosis, classified according to spinal cord radiotherapy dose.

There was no corresponding effect of posterior fossa or spinal cord dose on survival rates in the younger age group. Because of the close correspondence between the doses of radiotherapy to the whole brain and to the spinal cord, it was not possible to analyse the effects of doses to these two fields independently.

Table V summarises the results of significance tests on the survival curves for children in the two

**Table V** Effect on survival of extent of surgery (total excision *vs.* subtotal excision or biopsy); posterior fossa dose ($\geqslant 45$ Gy *vs.* $<45$ Gy) and spinal cord dose ($\geqslant 30$ Gy *vs.* $<30$ Gy): comparison of results of significance tests ($\chi^2$ on 1 df).

| *Age at diagnosis (years)* | | 0–4 | 5–14 |
|---|---|---|---|
| Effect of | Allowing for | | |
| Surgery | PF dose, SC dose | 2.78 | 0.10 |
| PF dose | Surgery, SC dose | 0.01 | 8.74* |
| SC dose | Surgery, PF dose | 0.00 | 3.43 |

PF = Posterior fossa.
SC = Spinal cord.
*$P<0.01$.

age groups, analysing the effects of the extent of surgery, posterior fossa dose, and spinal cord dose.

The overall actuarial rate of occurrence of spinal cord metastases in our series was 21% at 5 years. Among children aged 5–14 years the rate was 41% for those who received spinal cord doses of under 30 Gy compared with 14% for those with higher doses. The difference was statistically significant ($\chi^2 = 5.50$ on 1 df, $P < 0.05$). When posterior fossa dose was allowed for, the difference was no longer significant ($\chi^2 = 2.59$ on 1 df, $P > 0.1$). There was no evidence of an effect of spinal cord dose on the incidence of spinal metastases among younger children.

Figure 5 shows the survival curves for children classified according to whether they received chemotherapy as part of their initial planned treatment. The survival rate was higher among children who were given chemotherapy, but not significantly so ($\chi^2 = 2.83$ on 1 df, $P > 0.05$). When age, extent of surgery and posterior fossa radiotherapy dose were taken into account, there was no evidence of an effect on survival which could be attributed to chemotherapy. Because of the wide variety of combinations of drugs which were used during the period covered by this study, it was not possible to assess the effects of individual chemotherapy regimes.

**Figure 5** Actuarial survival curves for children of all ages combined, classified according to whether chemotherapy was included in initial planned treatment. (——) chemotherapy; ($n = 90$); (----) no chemotherapy ($n = 214$).

The neurosurgical centres were divided into 4 groups according to whether the total number of cases of childhood medulloblastoma treated by them during 1971–77 was in the range 1–5, 6–10, 11–20 or over 20. There was found to be no difference in survival rates between the four groups. The same classification was used for the radiotherapy centres, and again there was no difference in survival rates.

### Discussion

The present series of children was ascertained from the national cancer registration scheme, which is notified of over 90% of all cases of childhood cancer in Great Britain (Draper *et al.*, 1982); it is thus population based and nearly complete. The total number of 304 patients completing their initial planned course of radiotherapy is considerably larger than any previously reported in a detailed study of childhood medulloblastoma. There is known to be a risk of mortality for some years after diagnosis with this tumour; the minimum period of follow-up in our study is 5 years, with some patients having been followed up for as long as 12 years after diagnosis. The age distribution of this series was similar to that of a large series aggregated from a review of the literature (Choux *et al.*, 1982) except that ours contained a larger number of children aged 10 years and over. This discrepancy may be explained by the fact that many of the series reviewed were from children's hospitals and would be expected to contain a preponderance of younger patients.

The post-operative treatment administered to individual children with medulloblastoma during 1971–77 was in general not randomly allocated within a clinical trial. However, a close examination of the data relating to children treated at particular centres suggests very strongly that at any centre at any given time the doses of radiotherapy were standardised, though with a tendency at some hospitals for older children to be given higher doses. It appears that at the great majority of hospitals at any time chemotherapy was either included in the initial planned treatment for all children with medulloblastoma or for none, or was randomly allocated within clinical trials. Therefore, although the analyses of survival related to treatment should be interpreted with some caution, it seems unlikely that differences in survival rates between groups of children undergoing different treatment are attributable to selective bias in the allocation of treatment to different categories of patient.

From the results presented above, it is clear that the short-term prognosis is poorer for children aged under 5 years than for older children. However, in the longer term it appears that the survival rates for the two age groups are similar, and in the present series the 6-year survival rates for children who completed their treatment were 34% for those aged 0–4 years at diagnosis and 35% for those aged 5–14 years. Bloom *et al.* (1969) also found that long-term survival rates were similar for patients of all ages but that deaths tended to occur earlier among younger patients. The tendency for mortality to be more concentrated in a short period after diagnosis among younger patients has also been noted in children with neuroblastoma, another embryonal tumour of nervous tissue (Kinnier Wilson & Draper, 1974; Draper *et al.*, 1982).

In the past total excision of a medulloblastoma has generally been held to result in higher survival rates (Choux *et al.*, 1982). Among the children aged under 5 years in the present series, survival rates were considerably higher for those who had undergone total surgical removal of their tumours. In the older age group the extent of the surgery appeared to make little difference to survival rates, except that the survival rate for patients whose tumours were merely biopsied was lower. In 13/43 children whose surgery was limited to biopsy it was explicitly stated that the tumour was inoperable, and this presumably also applied in some other cases. Where excision of the tumour was attempted, many factors may have influenced the degree of removal, including tumour size and the extent of local spread. In particular no tumour involving the brain stem will have been totally removed. Brain stem involvement has been previously reported as an indicator of poor prognosis (Bloom *et al.*, 1969), and the 5-year survival rate in our series was indeed extremely low (1/13, 8%). However, it appears very likely that we failed to ascertain many cases of brain stem involvement; in our series 13/304 (4%) such cases were reported, whereas in a recent study of childhood medulloblastoma by the United Kingdom Children's Cancer Study Group, brain stem involvement was noted in 7/49 (14%) (C.C. Bailey, personal communication).

There was a clear advantage associated with a radiotherapy dose of at least 45 Gy to the posterior fossa in children aged 5 years and over, but higher doses apparently did not improve the prospects for survival for younger children. In a report on children aged under 5 years with brain tumours, Deutsch (1982) found very few survivors who received a dose of less than 40 Gy, but it is impossible to deduce the dose given to the patients with medulloblastoma in his series and for some patients the period of follow-up was very short. We found a tendency for higher doses to the posterior fossa to be associated with higher spinal cord doses, and this fact makes it difficult to determine the effect on survival of the spinal cord radiotherapy dose. Nevertheless when allowance was made for the effect of posterior fossa dose, the higher survival rate and lower incidence of spinal cord metastases amongst the older age group who received at least 30 Gy to the spinal cord both approached statistical significance.

There appeared to be little influence of chemotherapy on survival rates in our series but certain combinations of drugs may have a beneficial effect which it was not possible to detect in the present study because of the small number of children treated according to any one protocol. There is as yet no clear consensus amongst workers using standardised protocols on the effects of chemotherapy on survival in patients with medulloblastoma. Some studies have shown higher survival rates to be associated with chemotherapy, at least in some subgroups of patients (Berry *et al.*, 1981; Bloom *et al.*, 1969; Bloom, 1982*a,b*), while in others there has so far been no demonstrable benefit derived from the use of chemotherapy (Evans *et al.*, 1979; van Eys *et al.*, 1981).

In the great majority of children who completed their treatment for medulloblastoma and subsequently died, death was caused by the tumour, though a small number of exceptions has been identified. Two children, who had both undergone maintenance chemotherapy with BCNU, died with fibrosing changes in the lungs at 30 and 44 months after diagnosis, and were apparently tumour-free at the time of death. The fatal lung disease in these children was attributed to the use of BCNU (Bailey *et al.*, 1978) and this drug has since been widely demonstrated to cause pulmonary fibrosis. One child died from progressive cerebral necrosis of unknown cause 39 months after the diagnosis of medulloblastoma, and was found to be tumour-free at post mortem. Another patient developed acute undifferentiated leukaemia and died 27 months after diagnosis of the original tumour. There were no other cases of second primary neoplasms in the present series. However, in a series of childhood medulloblastoma survivors treated in earlier years (1940 onwards) and with a correspondingly longer period of follow-up, cases of basal cell carcinoma, osteosarcoma, fibrosarcoma, meningioma and carcinoma of the colon have been observed (Kingston *et al.*, in preparation). In a study of 58 patients who died more than 5 years after treatment of childhood medulloblastoma during 1940–70, 48 (83%) deaths were directly attributable to the tumour, 5 were due to second primary neoplasms and 5 were due to other causes including infections and accidents (J.E. Kingston, personal communication). In the present series there have so far been 14 patients who died over five years after diagnosis, with recurrent tumour being the underlying cause of death in all cases.

In conclusion, the improvement in survival rates for childhood medulloblastoma during the period under review may be largely attributed to the effects of higher doses of radiotherapy on older children. Any definitive statement on the advantages of chemotherapy must await the analysis and publication of the several recent and current clinical trials for this tumour. The only factor which appeared to influence the prognosis for younger children was the extent of surgical removal of the tumour. With recent advances in neurosurgical techniques, the possibility that more extensive surgery may compensate for lower doses of radiotherapy could be explored in a prospective study. Effective treatment of childhood medulloblastoma may then be achieved without the long term complications associated with high doses of radiation.

We thank the many consultants and general practitioners who provided the information on which this paper is based. We are grateful to the Office of Population Censuses and Surveys, the Information Services Division of the Common Services Agency of the Scottish Health Service, the General Register Office for Scotland, and regional cancer registries for providing copies of notifications of childhood cancer cases. We thank the National Health Service Central Register for notification of deaths and the "flagging" of survivors. We are grateful to Dr. L.M. Kinnier Wilson for providing data from the Oxford Survey of Childhood Cancers, to Mrs. E.M. Roberts for her part in collecting the medical records and for secretarial help and to Mrs. J. Welch for drawing the graphs. The Childhood Cancer Research Group is supported by the Department of Health and Social Security and the Scottish Home and Health Department. Collection of data was also supported by the Marie Curie Memorial Foundation.

### References

BAILEY, C.C., MARSDEN, H.B. & MORRIS JONES, P.H. (1978). Fatal pulmonary fibrosis following 1, 3-BIS (2-chloroethyl)-1-Nitrosourea (BCNU) therapy. *Cancer*, **42**, 74.

BERRY, M.P., JENKIN, R.D.T., KEEN, C.W., NAIR, B.D. & SIMPSON, W.J. (1981). Radiation treatment for medulloblastoma. A 21 years review. *J. Neurosurg.*, **55**, 43.

BLOOM, H.J.G. (1979). Recent concepts in the conservative treatment of intra-cranial tumours in children. *Acta Neurochir*, **50**, 103.

BLOOM, H.J.G. (1982*a*). Intracranial tumours: response and resistance to therapeutic endeavours, 1970–1980. *Int. J. Radiat. Oncol. Biol. Phys.*, **8**, 1083.

BLOOM, H.J. (1982*b*). Medulloblastoma in children: increasing survival rates and further prospects. *Int. J. Radiat. Oncol. Biol. Phys.*, **8**, 2023.

BLOOM, H.J.G., WALLACE, E.N.K. & HENK, J.M. (1969). The treatment and prognosis of medulloblastoma in children. A study of 82 verified cases. *Am. J. Roentgenol. Radium Ther. Nucl. Med.*, **105**, 43.

CHOUX, M., LENA, G., ALFONSI, S. & 13 others. (1982). Le Médulloblastome. *Neurochirurgie*, **28**, (suppl.) 1.

DEUTSCH, M. (1982). Radiotherapy for primary brain tumours in very young children. *Cancer*, **50**, 2785.

DRAPER, G.J., BIRCH, J.M., BITHELL, J.F. & 6 others. (1982). *Childhood Cancer in Britain: Incidence, Mortality and Survival.* Studies in medical and population subjects, No. **37**, HMSO, London.

EVANS, A.E., ANDERSON, J., CHANG, C. & 4 others. (1979). Adjuvant chemotherapy for medulloblastoma and ependymoma. In: *Multidisciplinary Aspects of Brain Tumour Therapy*, (Eds. Paoletti *et al.*). Elsevier-North-Holland, Amsterdam.

GEROSA, M., DI STEFANO, E., OLIVI, A. & CARTER, A. (1981). Multidisciplinary treatment of medulloblastoma: 5 year experience with the SIOP trial. *Child's Brain*, **8**, 107.

KINNIER WILSON, L.M. & DRAPER, G.J. (1974). Neuroblastoma, its natural history and prognosis: a study of 487 cases. *Br. Med. J.*, **iii**, 301.

MARSDEN, H.B. & STEWARD, J.K. (1976). *Tumours in Children* (2nd edition). *Recent Res. Cancer Res.*, **13**, 184.

VAN EYS, J., CHEN, T., MOORE, T., CHEEK, W., SEXAAR, C. & STARLING, K. (1981). Adjuvant chemotherapy for medulloblastoma and ependymoma using i.v. vincristine, intrathecal methotrexate and intrathecal hydrocortisone: a South West Oncology Group study. *Cancer Treat., Rep.*, **65**, 681.


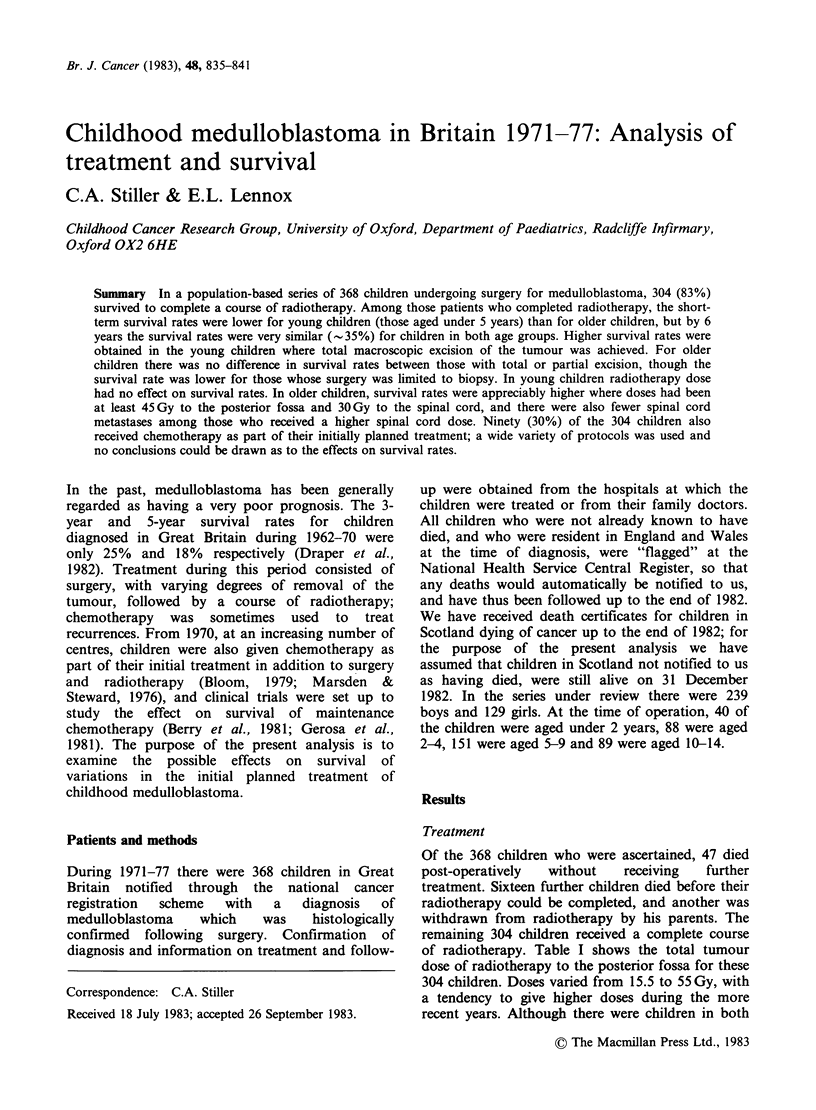

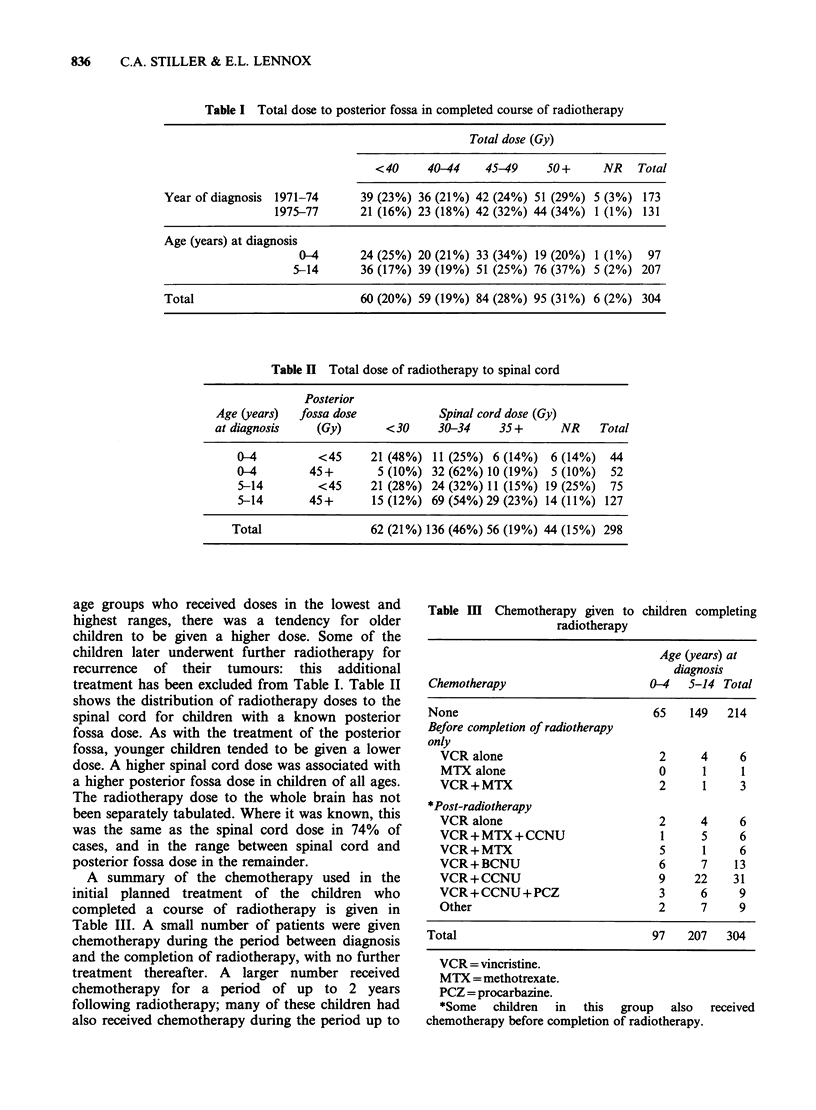

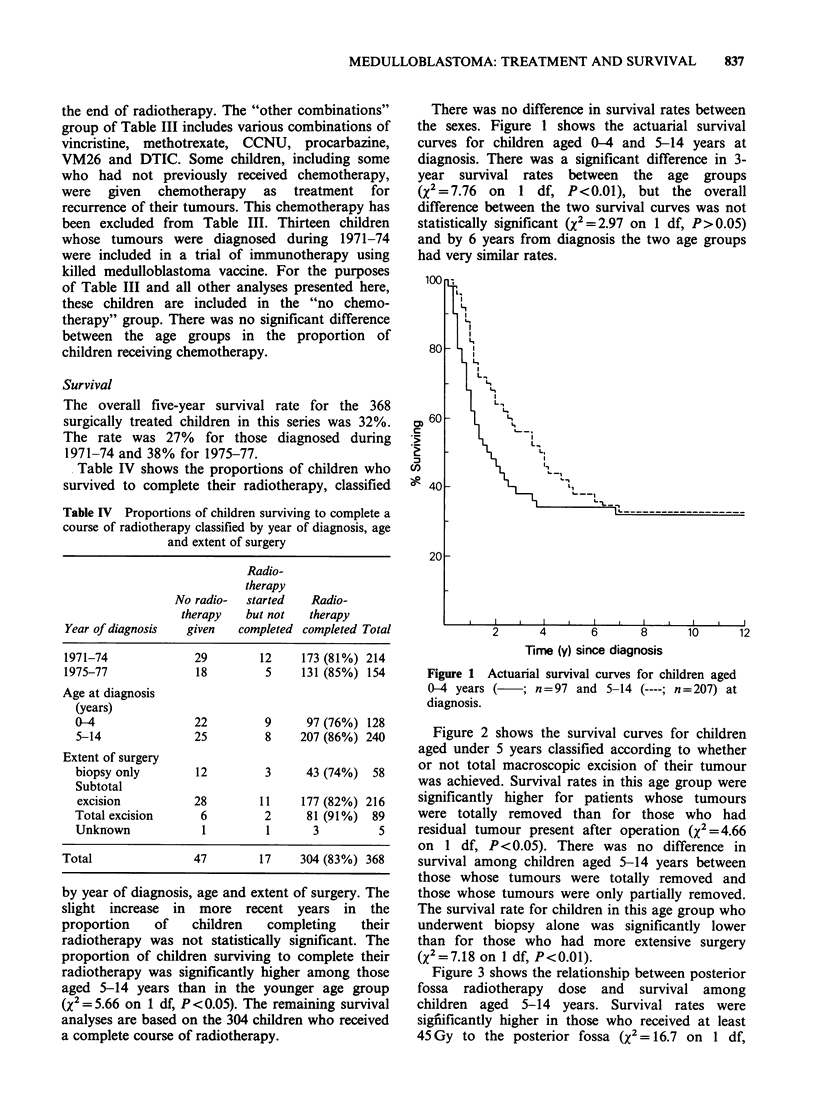

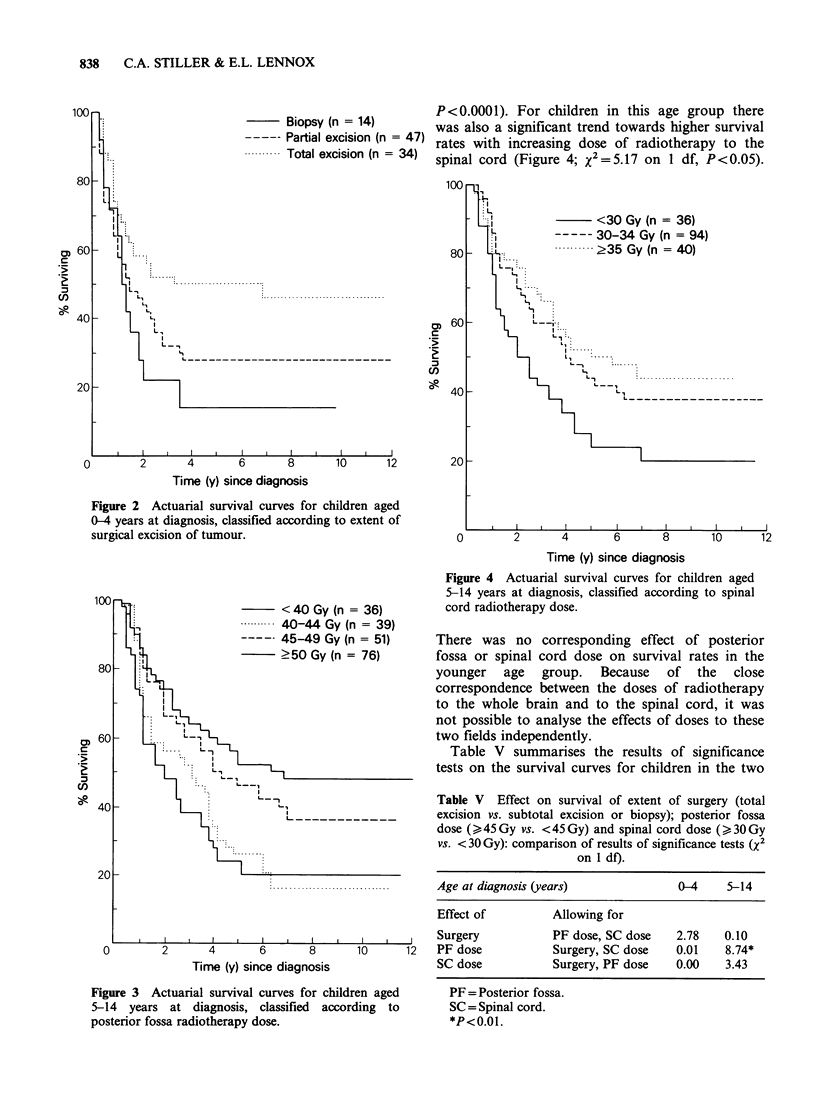

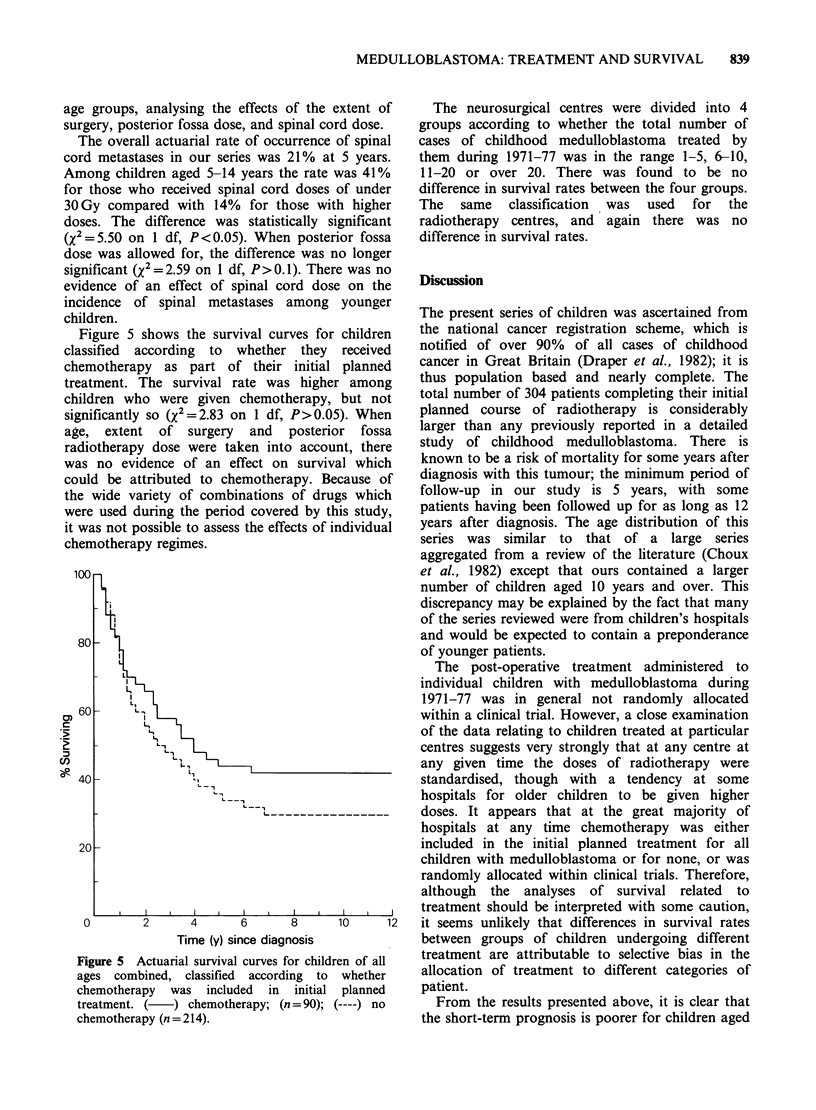

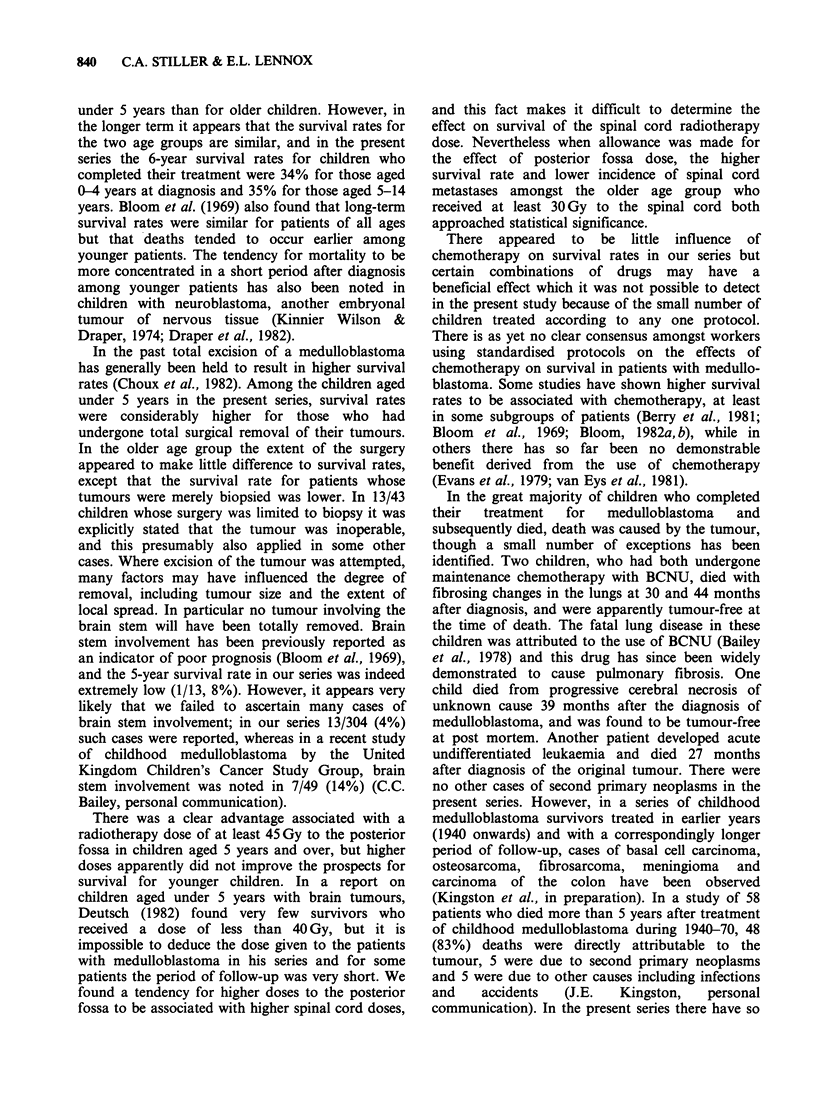

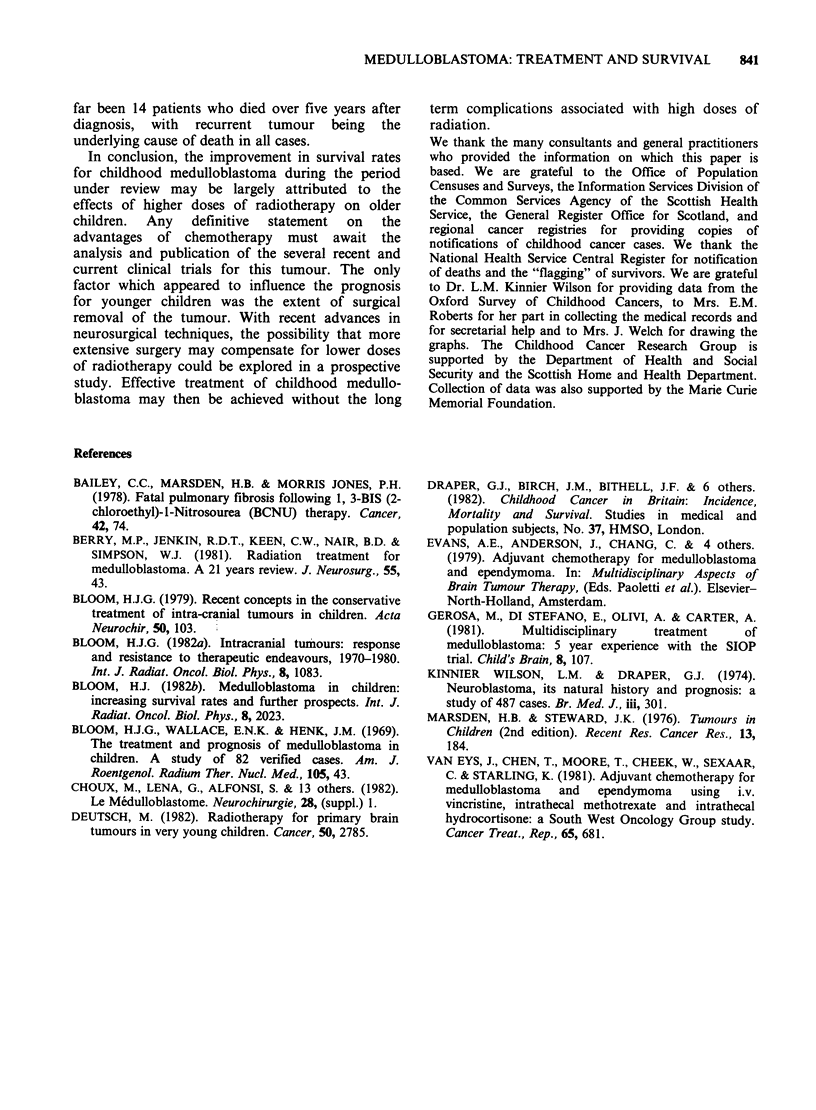


## References

[OCR_00689] Berry M. P., Jenkin R. D., Keen C. W., Nair B. D., Simpson W. J. (1981). Radiation treatment for medulloblastoma. A 21-year review.. J Neurosurg.

[OCR_00700] Bloom H. J. (1982). Intracranial tumors: response and resistance to therapeutic endeavors, 1970-1980.. Int J Radiat Oncol Biol Phys.

[OCR_00705] Bloom H. J. (1982). Medulloblastoma in children: increasing survival rates and further prospects.. Int J Radiat Oncol Biol Phys.

[OCR_00695] Bloom H. J. (1979). Recent concepts in the conservative treatment of intracranial tumours in children.. Acta Neurochir (Wien).

[OCR_00710] Bloom H. J., Wallace E. N., Henk J. M. (1969). The treatment and prognosis of medulloblastoma in children. A study of 82 verified cases.. Am J Roentgenol Radium Ther Nucl Med.

[OCR_00720] Deutsch M. (1982). Radiotherapy for primary brain tumors in very young children.. Cancer.

[OCR_00737] Gerosa M. A., di Stefano E., Olivi A., Carteri A. (1981). Multidisciplinary treatment of medulloblastoma: a 5-year experience with the SIOP trial.. Childs Brain.

[OCR_00743] Wilson L. M., Draper G. J. (1974). Neuroblastoma, its natural history and prognosis: a study of 487 cases.. Br Med J.

[OCR_00753] van Eys J., Chen T., Moore T., Cheek W., Sexauer C., Starling K. (1981). Adjuvant chemotherapy for medulloblastoma and ependymoma using iv vincristine, intrathecal methotrexate, and intrathecal hydrocortisone: a Southwest Oncology Group Study.. Cancer Treat Rep.

